# From heart to gut: Exploring the gut microbiome in congenital heart disease

**DOI:** 10.1002/imt2.144

**Published:** 2023-10-30

**Authors:** Yuze Liu, Yuan Huang, Qiyu He, Zheng Dou, Min Zeng, Xu Wang, Shoujun Li

**Affiliations:** ^1^ Pediatric Cardiac Surgery Centre, Fuwai Hospital, National Centre for Cardiovascular Diseases, State Key Laboratory of Cardiovascular Disease, Chinese Academy of Medical Sciences Peking Union Medical College Beijing China; ^2^ Department of Pediatric Intensive Care Unit, Fuwai Hospital, National Centre for Cardiovascular Diseases, State Key Laboratory of Cardiovascular Disease, Chinese Academy of Medical Sciences Peking Union Medical College Beijing China

**Keywords:** cardiovascular surgery, congenital heart disease, immunoinflammatory homeostasis imbalance, intestinal microbiology, neurodevelopmental injury

## Abstract

Congenital heart disease (CHD) is a prevalent birth defect and a significant contributor to childhood mortality. The major characteristics of CHD include cardiovascular malformations and hemodynamical disorders. However, the impact of CHD extends beyond the circulatory system. Evidence has identified dysbiosis of the gut microbiome in patients with CHD. Chronic hypoxia and inflammation associated with CHD affect the gut microbiome, leading to alterations in its number, abundance, and composition. The gut microbiome, aside from providing essential nutrients, engages in direct interactions with the host immune system and indirect interactions via metabolites. The abnormal gut microbiome or its products can translocate into the bloodstream through an impaired gut barrier, leading to an inflammatory state. Metabolites of the gut microbiome, such as short‐chain fatty acids and trimethylamine *N*‐oxide, also play important roles in the development, treatment, and prognosis of CHD. This review discusses the role of the gut microbiome in immunity, gut barrier, neurodevelopment, and perioperative period in CHD. By fostering a better understanding of the cross‐talk between CHD and the gut microbiome, this review aims to contribute to improve clinical management and outcomes for CHD patients.

## Highlights

- Children with CHD experience gut microbiome dysbiosis in their early life.
- The gut microbiome affects CHD via immune stimulation and microbiome metabolites, influencing immunity, gut barrier, and inflammation.
- The bidirectional interaction between the gut microbiome and CHD plays an important role in CHD's development, treatment, and prognosis.

## Graphical abstract

Children with congenital heart disease (CHD) experience gut microbiome dysbiosis in their early life. The gut microbiome affects CHD via immune stimulation and microbiome metabolites, influencing immunity, gut barrier, and inflammation. The bidirectional interaction between the gut microbiome and CHD plays an important role in CHD's development, treatment, and prognosis.

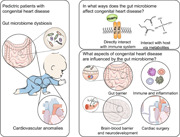

## INTRODUCTION

Congenital heart disease (CHD) is a common birth defect, afflicting approximately 2% of live newborns globally, and remains a significant contributor to infant morbidity and mortality [1, 2]. Despite advancements in medical technologies that have led to a decline in CHD‐related deaths, patients with CHD face an elevated risk of neurodevelopmental disabilities, immune dysfunctions, infections, and cancer [3, 4, 5, 6]. These potential complications have severe implications for the long‐term well‐being of CHD‐affected individuals and underscore the ongoing challenges in managing this complex congenital disease.

Research has revealed the vastness of the microbial population inhabiting the gastrointestinal tract, with estimates indicating that their numbers exceed 10^14^ and the genome is approximately 100 times more abundant than the human genome [7]. The complex interplay between the gut microbiome and its host extends far beyond the provision of essential nutrients. This cross‐talk is crucial to maintaining homeostasis, with studies suggesting its important role in the development of various physiological systems, including but not limited to the nervous, immune, and endocrine systems [8, 9, 10]. Moreover, the gut microbiome is involved in the progression of a spectrum of pathological conditions, such as intestinal epithelial barrier dysfunction (EBD) and systemic inflammation [11, 12].

In recent years, researchers have identified the substantial influence of gut microbiome composition and diversity on an individual's susceptibility to various diseases [13, 14, 15]. The gut microbiome has been demonstrated to have associations with various cardiovascular diseases [16, 17, 18, 19, 20, 21]. In our previous study, we provided the initial evidence that an aberrant gut microbiome, coupled with metabolic disruptions, was associated with immune imbalances and unfavorable clinical outcomes in neonates afflicted with critical CHD [22]. This finding underscores the significance of restoring an optimal gut microbiome to uphold host metabolic and immunological homeostasis. Additionally, some pathophysiological features of CHD, such as chronic hypoxia, are associated with changes in the composition and homeostasis of the gut microbiome [21, 23]. Cardiac operations, especially those requiring cardiopulmonary bypass (CPB) usage, are related to microbiome disorder and EBD, ultimately leading to the systemic inflammatory response postoperatively [16, 24, 25]. Furthermore, notable shifts in microbiome composition have been observed in patients receiving cardiac intensive care, which is relevant to clinical outcomes [26].

**Figure 1:**
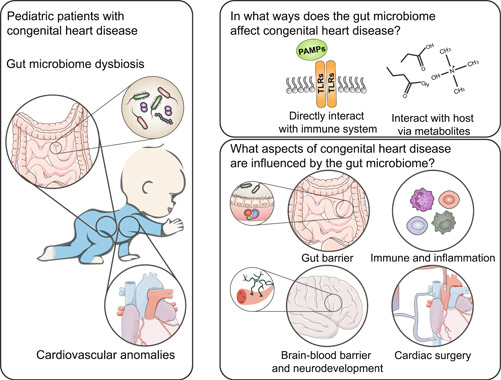
Relationship between CHD and the gut microbiome. The gut microbiome exerts its influence on the body through the production of metabolites and immune stimulations. In the development of CHD, the gut microbiome plays crucial roles in altering gut barrier function, modulating the immune system, and influencing the extent of injury and inflammation resulting from cardiac surgery. CHD, congenital heart disease; PAMP, pathogen‐associated molecular pattern; TLR, Toll‐like receptor.

Studies focusing on the gut microbiome and CHD have identified the significance of the gut microbiome in CHD. We believe that the meaningful role of the gut microbiome in CHD needs further emphasis and exploration. This review focuses on the role of the gut microbiome in pediatric immunity, gut barrier, neurodevelopment, cardiac surgery, and postoperative pediatric intensive care unit (PICU). We bring together the relationship of the gut microbiome in multiple aspects of CHD, aiming to understand the underlying mechanisms and implications of such associations with a focus on identifying potential therapeutic interventions and directions for future research (Figure 1).

## FORMATION AND DEVELOPMENT OF THE EARLY LIFE GUT MICROBIOME

The formation of the gut microbiome undergoes dynamic changes from fetal to adulthood. The *Firmicutes* to *Bacteroidetes* ratio (*F*/*B* ratio) varies at different stages of a lifetime: 0.4 in infants, 10.9 in adults, and 0.6 in older people [27]. Studies have demonstrated the presence of the microbiome in the meconium, which originated from uteri and was associated with gut colonization [28]. *Bacilli* and other *Firmicutes* are the dominant bacteria groups in meconium [29]. Similarly, the most abundant phyla in infants are *Firmicutes* and *Bacteroidetes*, followed by *Actinobacteria* and *Proteobacteria* [30]. The species diversity in infant fecal microbiomes is highest within the first 24 h after birth and decreases over the first‐week postpartum [31]. This change may be attributed to some microbiomes from maternal or other sources that do not colonize the newborn's digestive tract. In the early years of life, *Bifidobacteriaceae* typically increases and becomes the dominant microbiome in infant feces [32]. However, the proportion of phylotypes belonging to *Bifidobacterium longum* declines with age [33]. In healthy adult individuals, *Bacteroidetes* and *Firmicutes* exhibit the highest abundance [34].

The intrapersonal variation of the gut microbiome is more significant in children compared with adults [33]. During early life, various factors can influence the composition of the gut microbiome. Maternal obesity is linked to the richness of neonates' *Firmicutes* and elevated risk of becoming overweight [35]. A meta‐analysis pointed out that intrapartum antibiotic use, maternal overweight/obesity, and gestational weight gain were associated with reduced diversity in the infant gut microbiome [36]. Premature infants, in contrast to full‐term infants, exhibit higher levels of facultative anaerobic microbes and lower levels of strict anaerobes, such as *Bifidobacterium*, *Bacteroides*, and *Atopobium* [37]. The comparison of the fecal samples of infants and their mothers indicates that 72% of the species of early life microbiomes in vaginally delivered infants matches those microbiomes of their mothers, whereas this proportion is 41% in C‐section newborns [30]. The mother‐to‐child microbiome transmission via human milk is an important way for infants to gain microbiomes, such as *Bifidobacterium* and *Staphylococci* [38]. The human milk oligosaccharides (HMO) may partially contribute to the microbiological variation and the gut barrier function for infants fed with breast milk [39]. Certain microorganisms, including *B. longum* subsp. *infantis*, *Bacteroides fragilis*, and *Bacteroides vulgatus* strains, can easily metabolize HMO [40, 41]. Additionally, formula feeding leads to alterations in the microbiome associated with obesity, potentially attributed to the higher relative abundance of *Lachnospiraceae* at 3–4 months after birth [42]. It is worth noting that the gut microbiome of breastfed infants originates not only from breast milk itself but also from the skin of the areola. It was reported that breastfed infants acquired 27.7% of their bacteria from breast milk and 10.3% from the areolar skin [32]. The induction of solid food is associated with an elevated abundance of bacteria producing butyrate and a reduced amount of *Enterobacteriaceae* and *Staphylococcus* [43]. Another factor affecting the infant microbiome is antibiotic use, which has been linked to the reduction of microbiome richness and diversity [44]. The administration of intrapartum antibiotic use influences microbial communities and antimicrobial genes in the gut microbiome of infants [45]. A randomized trial examining broad‐spectrum antibiotics against sepsis indicated a decreased abundance of *Bifidobacterium* and an increased abundance of *Klebsiella* and *Enterococcus* [46].

## MICROBIOME METABOLITES AND CARDIOVASCULAR DISEASES

**Figure 2:**
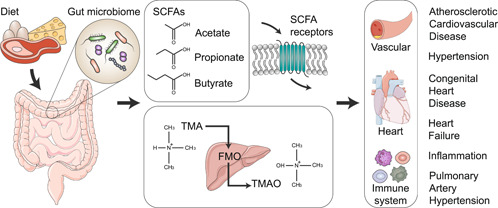
Associations between microbiome metabolites and cardiovascular diseases. Short‐chain fatty acids (SCFAs) are generated through the fermentation of indigestible polysaccharides by the gut microbiome and act on specific SCFAs receptors. Trimethylamine (TMA), a metabolite of the gut microbiome, undergoes an oxidation process in the liver via flavin‐containing monooxygenases (FMOs) and is subsequently converted into trimethylamine *N*‐oxide (TMAO). These metabolites exert both direct and indirect effects on the cardiovascular system, and they have been associated with certain cardiovascular diseases.

Microbiome metabolites play important roles in various physiological processes and have been found to have significant effects on cardiovascular diseases (Figure 2) [47]. Some of the metabolites include short‐chain fatty acids (SCFAs), trimethylamine (TMA), and trimethylamine *N*‐oxide (TMAO). SCFAs are fatty acids with less than six carbon atoms, produced by saccharolytic fermentation of undigested carbohydrates, mainly consisting of acetate, propionate, and butyrate [48, 49]. SCFAs can target signal receptors on various cells within the cardiovascular system as well as other systems, like, the immune system and adipose tissue [50]. SCFAs regulate blood pressure by acting as the ligand to the G‐protein coupled receptors (GPCRs), including GPR41, GPR43, GPR109A, OR51E2, and OR51E1 [51]. The connection between SCFAs and GPCRs affects vascular endothelial cells and immune cells, leading to increased peripheral vascular resistance, vascular remodeling, and renal sodium absorption [51]. SCFAs have a protective effect on atherosclerotic cardiovascular disease (ACVD). Feeding apolipoprotein E knockout‐deficient mice with propionate inhibits angiotensin II‐induced atherosclerosis [52].

The gut microbiome breaks down compounds containing TMA, such as red meat, resulting in the production of TMA. This TMA can then undergo further oxidation in the host liver to generate TMAO [53, 54]. Elevated plasma TMAO level has been associated with an increased incidence of all‐cause cardiovascular mortality and major adverse cardiac and cerebrovascular events [55]. TMAO has a proatherogenic property, typically by enhancing vascular endothelial cell inflammation, inhibiting reverse cholesterol efflux, and promoting platelet aggregation and thrombosis [54]. TMAO activates NLRP3 inflammasome and then triggers vascular inflammation [56]. Moreover, a high circulating TMAO level is related to poor prognosis in various types of pulmonary arterial hypertension (PAH), including PAH associated with CHD [57].

The gut microbiome affects the metabolism of bile acids, especially secondary bile acids. The primary bile acids are synthesized in the liver and then conjugated with glycine or taurine to form conjugated bile acids. The conjugated bile acids are secreted into bile and then into the gut, where they are deconjugated by the gut microbiome and transformed into secondary bile acids. The primary bile acid‐to‐secondary bile acid ratio may be an important influencing factor in cardiovascular diseases [58]. Patients with chronic heart failure have a decreased primary bile acids level and an increased secondary bile acids level [59]. Bile acids are proven to be associated with lipid metabolism, glucose metabolism, and inflammation, which may be the reason why bile acids metabolism is important in cardiovascular disease [60].

Numerous studies have shown that the disruption of the host gut microbiota can significantly impact the occurrence and development of various cardiovascular diseases. Alterations in the maternal gut microbiome, characterized by a reduction in alpha diversity and distinctive microbiome composition, along with changes in plasma metabolites, were observed to correlate with an increased risk of offspring CHD [61]. The decreased composition of certain probiotics may affect fetal development through impaired gut barrier, chronic inflammation, and insulin resistance [61]. The disorder of gut microbiome and metabolism was observed in patients with CHD and heart failure, which was characterized by decreasing microbiome diversity, richness, and downregulation of retinol metabolism [62]. The serum level of bile acids in patients with repaired Tetralogy of Fallot (TOF) has a positive correlation with indexed right ventricular end‐diastolic volume, which may suggest impaired liver function due to right ventricular dysfunction and systemic congestion [63]. However, it is worth noting that the impact of gut microbiota dysbiosis on the phenotypes of these diseases related to CHD has not yet been systematically and comprehensively summarized.

## CHD, GUT MICROBIOME, AND IMMUNITY

The immune system starts to develop in the fetus alongside the formation of the heart and intestine. Functional thymus and the production of circulating T cells commence at 10–11 weeks of gestation, and B cells can be detected in the spleen between the 12th and 23rd weeks of gestation [64]. The birthing process exposes the infant to a wide range of microorganisms, initiating the colonization of beneficial bacteria on the skin and in the gastrointestinal tract. Studies have highlighted the role of the immune system in CHD, which is primarily characterized by systemic inflammation and immune alterations [65].

CHD patients have shown the presence of circulating markers, primarily cytokines. Fan et al. reported the observation of immune activation in patients with CHD, which was evident through an elevation in inflammatory cytokines and a reduction in anti‐inflammatory cytokines [66]. These cytokine levels returned to normal following transcatheter CHD treatment [66]. Various studies have reported elevated levels of tumor necrosis factor‐alpha (TNF‐α) and interleukin‐6 (IL‐6) in CHD patients [67, 68, 69]. It has been established that there is a positive correlation between the levels of growth differentiation factor‐15, β2‐microglobulin, and the severity of chronic heart failure [70]. Additionally, the elevated circulating inflammatory cytokines may be associated with diaphragm dysfunction and restrictive ventilation disorder in CHD patients [71]. Other circulating markers, such as IL‐1, IL‐8, plasma endotoxin, and ghrelin, also indicate systemic inflammation of CHD [65].

Patients with CHD, especially cyanotic CHD, experience persistent exposure to hypoxia‐caused myocardial stress, which triggers the activation of the innate immune system at the cellular level, leading to the activation of proinflammatory cells. [72]. The elevated Neutrophil‐Lymphocyte Ratio (NLR) has been identified in patients with CHD [73, 74]. Other components of the innate immune system, including monocytes, natural killer cells, and mast cells are also activated [65]. T cells are key components of the adaptive immune response as they possess the ability to recognize and selectively target pathogens. Upon maturation, T cells circulate throughout the body, patrolling the blood and lymphatic system in search of foreign invaders. As T cells undergo maturation within the thymus, a byproduct known as T cell receptor excision circles (TRECs) is produced, serving as biomarkers of lymphopoiesis [75]. Compared with the normal population, a reduced level of TRECs in children with CHD has been observed, and a combination of reduced and premature TRECs copies was associated with infection‐caused hospitalization [76]. However, no correlation was found between the TRECs level and the severity of CHD [76].

Surgical repair of cardiac anomalies usually occurs within the first several years of a lifetime. Thymus removal is a procedure often performed during CHD operations to enhance the visibility of the heart and facilitate the surgical intervention, while this process may also have implications for the ongoing immune development of children with CHD [65, 77]. Early thymectomy in CHD patients is associated with changes in the T cell compartment, including decreased total T cells, CD4^+^ T cells, CD8^+^ T cells, and a reduced diversity of the T cell receptor [78]. The peripheral naive T cell subset in thymectomy patients exhibits similarities to the immune profile seen in elderly individuals with thymic involution, characterized by decreased counts of CD4^+^CD45RA^+^CD62L^+^ T cells [79]. Besides, elevated levels of the antinuclear antibody and the antineutrophil cytoplasmic antibody are correlated with a higher percentage of CD4^+^ memory T cells after early thymectomy [80].

The pathogen‐associated molecular patterns (PAMPs), which consist of surface layer proteins, flagella, pili, and capsular polysaccharides of the microbiome, are specifically identified by pattern recognition receptors (PRRs) [81]. Typically, continuous communication does not result in inflammation; on the contrary, it enhances host immune function [82]. However, when commensal microorganisms are misidentified, a consistently activated immune response occurs, which can be detrimental to the host [83]. The gut microbiome can substantially affect host immune homeostasis in early life, play an essential role in the development of postnatal intestinal endotoxin tolerance, and have an impact on immune cells [84, 85]. A higher relative abundance of *Bifidobacteria* has been reported to be associated with improved CD4^+^ T‐cell responses and vaccine memory [86]. SCFAs have a systemic anti‐inflammatory effect by upregulating anti‐inflammatory while downregulating proinflammatory cytokines [87]. SCFAs regulate the mitogen‐activated protein kinase and nuclear factor kappa‐B (NF‐κB) signaling pathways through the activation of free fatty acid receptors type 2/3, GPR109A, as well as the inhibition of histone deacetylases [88]. SCFAs produced by commensal microbiomes facilitate the extrathymic generation of regulatory T cells (Tregs) and ameliorate the development of intestinal inflammation [89, 90]. Another metabolite, 12,13‐diHOME, a monohydroxy fatty acid, influences the development of Tregs in early life [91].

## HYPOXIA AND HYPOPERFUSION AFFECT THE GUT BARRIER

A symbolic pathophysiological characteristic of CHD is chronic hypoxia. Patients with CHD may experience hypoxemia or tissue hypoxia due to structural abnormalities in their hearts or large vessels. The extent of hypoxia mainly depends on the type and severity of the cardiac defect, including shunting (e.g., patent ductus arteriosus), blood flow obstruction (e.g., coarctation of the aorta), inadequate pumping (e.g., dilated cardiomyopathy), and cyanotic CHDs (e.g., TOF).

It is well known that hypoxia is associated with inflammation. The myocardial response to chronic hypoxia parallels that of bacterial infection, leading to the generation of molecules, such as reactive oxygen species (ROS) [72]. Hypoxia induces ROS production at mitochondrial Complex III, activating inflammatory pathways and stimulating the production of proinflammatory cytokines [92]. Despite the lower oxygen concentration in the digestive tract, especially the large intestine, pathophysiologic hypoxia still causes intestinal inflammation and gut barrier injury [23, 93]. Under inflammatory conditions, oxidative stress generated by neutrophils results in the disruption of interendothelial junctions and facilitates the migration of inflammatory cells across the endothelial barrier, contributing to the clearance of pathogens and leading to tissue injury [94]. Children with CHD face an increased risk of intestinal barrier injury. The overgrowth of *Enterococcus* in CHD neonates has been associated with gut barrier dysfunction and stimulated inflammatory responses, evidenced by increased levels of intestinal fatty acid binding protein (FABP) and d‐lactate [22]. Steck et al. indicated *Enterococcus* compromises the gut barrier via gelatinase, a metalloprotease secreted by *Enterococcus faecalis* [95].

**Figure 3:**
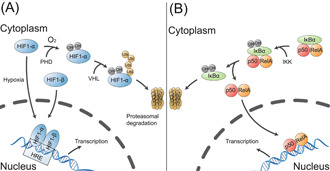
Regulation of HIF and NF‐κB. (A) Regulation of HIF in hypoxic and normoxic conditions. In hypoxic conditions, HIF‐α stabilizes and forms a complex with HIF‐β. This HIF complex translocates to the nucleus, binds to hypoxia response elements (HREs), and activates genes that help the cell adapt to low oxygen. HIF‐α is hydroxylated by prolyl hydroxylases (PHDs) under normoxic conditions. The hydroxylated HIF‐α is then catalyzed by the von Hippel–Lindau (VHL) protein, leading to its subsequent degradation through the proteasome pathway. (B) The canonical activation process of NF‐κB. NF‐κB is located in the cytoplasm and binds with the inhibitory protein IκBα. When IκB kinase (IKK) is activated, the IκBα is phosphorylated and degraded via the ubiquitination pathway. With the degradation of IκBα, the p50, and RelA dimer translocates to the nucleus, where it activates the transcription of multiple target genes. HIF, hypoxia‐inducible factor; NF‐κB, nuclear factor kappa‐B.

The hypoxia‐inducible factor (HIF) is a protein dimer that orchestrates the expression of several genes in adaptive responses to oxygen deprivation (Figure 3A). As the key molecule in adapting to chronic hypoxia, HIF‐1α upregulated glucose utilization and downregulated fatty acid utilization to maintain adenosine triphosphate supply and cardiac function [96]. Mutations in EPAS1 (gene encoding HIF‐2α) have been identified in patients with CHD residing in high‐altitude regions [97]. These EPAS1 mutations are associated with angiogenesis, demonstrating adaption to chronic hypoxia.

One of the significant roles of HIF lies in maintaining gut barrier function via regulating molecules related to the tight junction (claudin‐1) and epithelial surface repair (Trefoil factor‐3) [98]. The microbiome maintains the gut barrier by generating metabolites and interacting with PRR in the gut mucosa, thus preserving gut homeostasis [99]. Healthy diets are metabolized by microbiomes to yield beneficial metabolites that interact with particular receptors located on the membrane or nucleus, regulating HIF and enhancing barrier function [100]. For example, microbiome‐derived butyrate stabilizes HIF by inhibiting HIF prolyl hydroxylases [101].

Chronic hypoxia increases the level of ROS and is also associated with the activation of NF‐κB [102]. NF‐κB exists as a heterodimer of p50 and RelA subunits. When it is activated, the NF‐κB dimer is translocated into the nucleus and regulates the transcription of certain inflammatory genes (Figure 3B) [103]. The gut microbiome can affect NF‐κB‐mediated inflammatory pathways. *Streptococcus salivarius* inhibits the activation of the NF‐κB and its downstream inflammatory cytokines in intestinal epithelial cells [104, 105].

**Figure 4:**
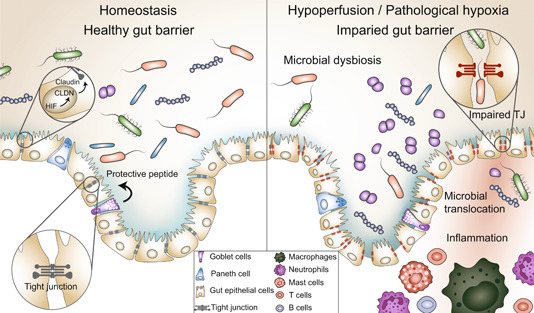
Pathological hypoxia/hypoperfusion induces microbiome dysbiosis and gut barrier dysfunction. Pathological hypoxia/hypoperfusion can result in an alteration of gut barrier integrity. The protective peptide in the mucus barrier and the tight junction between epithelial cells are important components for maintaining gut homeostasis. A weakened gut barrier may facilitate the translocation of bacteria and/or their byproducts from the gut lumen into the surrounding tissues, triggering an amplified inflammatory response. CLDN, claudin; HIF, hypoxia‐inducible factor; TJ, tight junction.

Similar to hypoxemia, reduced gut perfusion is also a risk factor for intestinal EBD, resulting in increased uptake of lipopolysaccharide (LPS) or other PAMPs [106, 107]. Impaired mucosal immunity and weakened intestinal barrier can lead to the translocation of bacteria and/or their products from the intestinal lumen into adjacent tissues, triggering an amplified inflammatory response and subsequent injury to the mucosal epithelium (Figure 4). CHD represents a risk factor for necrotizing enterocolitis (NEC) due to gut hypoperfusion. Reduced gut perfusion initiates inflammation, which, in turn, triggers secondary vasoconstriction that worsens gut hypoperfusion, thereby establishing a cycle of hypoperfusion and inflammation [108]. In patients with right heart dysfunction, systemic veinous congestion leads to intestinal edema and facilitates the endotoxin translocation from the intestinal tract into the bloodstream [109, 110].

## GUT MICROBIOME AND NEURODEVELOPMENT OF CHILDREN WITH CHD

A concern in CHD patients is the occurrence of neurodevelopmental disability (NDD). NDD is observed in nearly half of the children who have undergone cardiac intervention, leading to various challenges, such as cognitive dysfunction, attention and hyperactivity difficulties, motor functioning deficits, impaired language and communication skills [111]. NDD begins at gestation, as the complex circulation of CHD impacts the blood delivery to the fetal brain. Take transposition of the great arteries (TGA) as an example, the fetal brain of patients with TGA receives venous blood with decreased oxygen and metabolic substrates. TGA fetuses had lower brain‐to‐body ratios and higher cerebellar‐to‐total brain ratios compared with normal fetuses with similar gestational age and body growth, indicating immature brain development [112]. Risk factors such as prematurity, chronic hypoxia, and surgical interventions likely have a cumulative effect on the progression of NDD from the fetal period through childhood, with potential persistence into school age and even adulthood [113, 114]. Hemodynamically impaired CHD children have deficiencies in skills related to gross motor, fine motor, and language [115]. Moreover, children who underwent CHD operation with CPB have worse performance for cognition and fine motor skills [116].

The mechanisms underlying brain injury in CHD patients have not been fully studied. Tissue hypoxia and systemic inflammation can lead to the dysfunction of the blood–brain barrier (BBB) and subsequent injury to the nervous system, as indicated by the presence of the phosphorylated form of high molecular weight neurofilament protein [117]. Researchers have attempted to identify morphological differences between the brains of CHD patients and those of healthy individuals. It has been found a delayed brain maturation and a smaller gestational age‐ and weight‐adjusted total brain volumes in fetuses with CHD [118]. Additionally, children with CHD have smaller hippocampal volumes and surface area, and these morphologic alterations are correlated to executive function [119].

Delayed brain development increases susceptibility to acquired white matter injury (WMI). A cohort study involving fetuses/neonates with TGA who underwent preoperative magnetic resonance imaging (MRI) scans revealed that the brain volume of the fetus was associated with acquired WMI after birth [120]. The immaturity of the brain increases the write matter vulnerability to hypoxic‐ischemic brain injury [121, 122]. Early in the 1990s, acquired neuropathology, such as hypoxic‐ischemic lesions and intracranial hemorrhage, was found to be related to CPB procedures involving hypothermic total circulatory arrest lasting longer than 40 min [123]. The CPB‐induced oligodendrocyte dysmaturation and microglial expansion have been observed in a porcine model, suggesting brain damage [124]. Preterm infants exposed to general anesthesia are at an increased risk of brain abnormalities, as evidenced by findings from postoperative MRI scans [125].

The gut microbiome is involved in neurodevelopment in early life and the integrity of BBB, which contributes to NDD in patients with CHD. The gut microbiome can influence BBB integrity through microbe‐stimulated cytokines or metabolites derived from gut microbiomes [126]. Germ‐free mice exhibit higher BBB permeability, which begins with intrauterine life and maintains adulthood. However, exposure to a pathogen‐free gut microbiome has been shown to restore the permeability of BBB and enhance the expression of tight junction proteins, such as occludin and claudin‐5 [127]. The neuroprotective effect of the gut microbiome can be mediated by metabolites. Microbiomes that produce abundant SCFAs, such as *Clostridium tyrobutyricum*, have been found to improve BBB integrity in mice [127]. Moreover, TMAO has been shown to enhance the integrity of BBB, protect BBB from inflammatory injury, and improve cognitive function through the tight junction regulator Annexin A1 [128]. Another protective metabolite is *p*‐cresol glucuronide, the gut metabolite of amino acids tyrosine and phenylalanine. This metabolite promotes BBB integrity by antagonizing the LPS receptor TLR4 [129].

The gut microbiome has a significant impact on neuroimmune during brain development. Signals generated from the maternal microbiome may shape the development of fetal microglia [130, 131]. The microbiome metabolites have been shown to influence microglia maturity and functioning [132]. Acetate has been identified as an essential SCFA for microglia maturation and for maintaining their homeostatic metabolic state [133]. Moreover, maternal perinatal probiotic intake leads to an alteration of offspring's astrocyte metabolism, marked by the increased expression of prefrontal cortex PFKFB3 [134].

## GUT MICROBIOME AND PEDIATRIC CARDIOVASCULAR SURGERY

Cardiovascular surgery leads to significant alterations in the gut microbiome. Aardema H et al. analyzed fecal samples from patients who underwent coronary artery bypass graft and/or valve surgery [26]. The 16s RNA gene sequencing identified a significant increase of pathobionts (e.g., *Eggerthella*, *Enterococcus*, *Rothia, and Peptococcus*) and a decrease in strictly anaerobic SCFA‐producing gut bacteria (e.g., *Anaerostipes*, *Faecalibacterium*, *Blautia*, and *Roseburia*). The imbalance of the gut microbiome during the perioperative period may be associated with patients' clinical features and postoperative complications. The postoperative dysbiosis of the gut microbiome and metabolic abnormalities can impact patients' susceptibility to cardiac surgery‐associated acute kidney injury (AKI) [135, 136]. Analysis has identified *Escherichia coli*, *Rothia mucilaginosa*, and *Clostridium innocuum* as AKI‐related microbiomes [136]. Similarly, by analyzing fecal samples from cardiac surgery patients with postoperative pseudopsia, the increased count of *Staphylococcus* and *Pseudomonas* were identified [137].

CPB is a crucial and challenging technique employed in cardiac surgery to temporarily assume cardiac and pulmonary functions, ensuring continuous blood flow and oxygen supply. Most CHD surgery involves CPB, while CPB introduces extra risks to the surgery, such as ischemia‐perfusion injury (IRI) [138]. SCFAs have demonstrated the ability to improve cardiac function after IRI [48]. Propionate can act as a protector against myocardial IRI by alleviating the increased Angiotensin II levels through GPR41 [139]. Treatment with butyrate has been shown to significantly improve myocardial IRI via a gut–brain neural circuit, which can be inhibited by subdiaphragmatic vagotomy [140]. Other cardioprotective metabolites including Urolithin B, protect against IRI via the p62/Keap1/Nrf2 signaling pathway [141].

CPB is known to induce inflammation, both at the intestinal and systemic levels [142]. In the early stages following CHD operation, intermediate monocytes exhibit increased levels of CD64, TLR2, and TLR4, which may be associated with impaired postoperative recovery and organ dysfunction [143]. The classical perioperative factor NLR serves as a reflection of systemic inflammation and has been correlated with the composition of the gut microbiome [73, 74]. Elevated NLR is often a risk factor for unfavorable outcomes of CHD operation, including prolonged ventilation duration, extended ICU and hospital stay after TOF repair, reduced cardiac output, and worse single ventricular physiology after the bidirectional Glenn procedure [74, 144, 145, 146]. Compared with pediatric patients without cardiac surgery (without CPB), children who received CHD operation (with CPB) exhibit a greater relative abundance of *Actinobacteria* and *Proteobacteria*, as well as significant intestinal barrier dysfunction [16]. An animal experiment further revealed CPB‐mediated dysbiosis of the gut microbiome, SCFAs levels reduction, inflammatory cytokines expression, and EBD [147]. EBD is a important contributor of CPB‐induced systemic inflammation. The dysregulation of claudin‐2 and claudin‐3 after CPB may suggest impaired tight junction and elevated intestinal permeability [147]. However, the loss of gut barrier function seemed to begin preoperatively, marked by the elevated concentration of intestinal FABP2 [148]. Pediatric patients with CHD experienced a disrupted microbiome at baseline, which includes large proportions of proinflammatory, LPS‐expressing bacteria and reduction of gut health‐promoting bacteria [16]. These disruptions may be attributed to chronic hypoxia associated with CHD, and their severity may be exacerbated by subsequent CPB procedures.

Risk factors for impaired gut barrier not only include abnormal circulation, hypoxemia, and poor intestinal perfusion, but also include surgery‐related impairment. Studies have pointed out that children who underwent CHD surgery exhibit abnormal gut permeability [149]. The impaired permeability leads to postoperative endotoxemia, systemic inflammation, and organ dysfunction. Several pathways are activated after the CHD operation, including pathogen‐sensing pathways, antigen‐processing pathways, and immune‐suppressing pathways [150]. The Fontan procedure, a palliative intervention for complex CHD, creates a connection between the right atrium and the pulmonary arteries and redirects the oxygen‐depleted blood directly to the lungs without passing through the impaired right ventricle. Fontan circulation significantly improves the survival of certain CHD patients; however, it has several serious long‐term complications, such as protein‐losing enteropathy (PLE). Fontan‐PLE may be related to elevated resistance of mesenteric vasculature [151]. Patients with Fontan‐PLE exhibit abnormal intestinal permeability and elevated systemic proinflammatory cytokines, marked by intestinal FABP, TNF‐α, and IL‐6 [152].

Postoperative antibiotic use reduces both the richness and diversity of the gut microbiome and increases the relative abundance of *Enterococcus* and *Lactobacillus* [153]. Compared with preoperative antibiotic use, postoperative antibiotic use continuously impacts the gut microbiome, which may need an antibiotic withdrawal of more than 2 weeks to diminish.

## DISCUSSION

While numerous studies have elucidated the critical role of the gut microbiome in various aspects of life, including growth, aging, and various diseases, there is a paucity of research focused on CHD. It is evident that there exists a bidirectional cross‐talk between CHD and the gut microbiome. Nonetheless, the detailed interplay has primarily been explored through research conducted in other related fields, particularly studies on pediatric and adult cardiovascular surgery.

Another important CHD‐relevant field is prematurity. Although CHD and prematurity are distinct conditions, neonates with CHD are more likely to be born prematurely, and preterm neonates have increased possibilities of comorbidities with CHD [154]. CHD infants and premature infants share certain similarities, such as respiratory challenges, extended hospital stays, proinflammatory environment, immature immune system, and intestinal dysbiosis [155].

Preterm infants encounter sustained inflammatory challenges since birth. In terms of the immune defense, preterm infants primarily rely on nonspecific innate immunity, the functionality of T cell responses, including those mediated by T helper cells responsible for regulating inflammation, may be less effective. [156]. The immature hearts of preterm infants, when exposed to systemic inflammation, may experience perturbations in epigenetic modifications of genes involved in cardiac development [157]. In premature infants, the intestinal environment fosters the expansion of facultative anaerobes due to several factors, such as patchy mucous layer, increased permeability of tight junctions, and reduced antimicrobial peptides [158]. Dysbiosis of the gut microbiome may be associated with various pathological conditions. Take NEC as an example. Infants with NEC tend to exhibit a significantly lower relative abundance of *B. longum* and a higher relative abundance of *Enterobacter cloacae* in their gut microbiome profiles [159]. Premature infants have a high susceptibility to NEC due to the challenges their immature gut faces in managing enteral feeding and establishing a healthy bacterial environment [160]. In patients with critical CHD, there is also a noted decrease in the proportion of probiotics and an increase in the proportion of opportunistic pathogens [22]. Several studies have reported that the administration of probiotics can effectively reduce inflammation of preterm infants, resembling their gut environment to that of full‐term infants [161, 162, 163]. The above evidence underscores the importance of future research aimed at investigating microecological therapies targeting probiotic metabolites to improve many clinical phenotypes in children with CHD.

Despite the microbiome homeostasis in the gut, the microbiome in other systems is also worth investigating. For instance, oral microbiome homeostasis of CHD patients. Patients with CHD and caries have elevated abundance of *Fusobacterium*, *Prevotella*, *Capnocytophaga*, and *Oribacterium*, compared to healthy individuals [164]. It was reported that the immune dysregulation, production of proinflammatory cytokines, and progressive inflammation due to periodontal pathogens were responsible for ACVD [165]. Preventing oral microbiome dysbiosis may serve as an effective approach to reduce the risk of systemic inflammation and infection. Maternal microbiome, as they can affect offspring microbiome, should also be valued in research related to CHD. It has been identified that the maternal microbiome is associated with neurodevelopment, immunity, and inflammation [166, 167]. During pregnancy, microbes derived within the maternal gut are captured by dendritic cells and translocated to the fetus, then participate in the development of the offspring [168]. Furthermore, the maternal microbiome also plays a significant role in pregnancy‐related diseases, such as hypertensive disorder of pregnancy and insulin resistance, increasing the risk for fetal development and potential teratogenic effect [61, 169].

Several research gaps persist in our understanding of the relationship between the gut microbiome and CHD. While various studies have identified the direct involvement of the gut microbiome in cardiovascular diseases, limited research provides direct evidence of its role in CHD. The oral microbiome and maternal microbiome should also be valued as they play key roles in CHD occurrence and progression. The direct impact of metabolites should be carefully investigated in various aspects of CHD, such as growth, neurodevelopment, and immune development. Cells and molecules associated with inflammation, such as immune cells, cytokines, HIF, and NF‐κB within this cross‐talk are also essential areas of exploration. The relationship between the gut microbiome and noncoding RNAs, such as micro‐RNAs also warrants comprehensive examination. Existing studies have identified micro‐RNAs as regulators in both cardiovascular diseases and the gut microbiome. Several biological processes associated with CHD, such as cardiac development, myocardial protection during hypoxia, and adaptive right ventricular hypertrophy in response to PAH, have been linked to certain micro‐RNAs [170].

The therapeutic potential of the gut microbiome in the treatment of CHD is also a promising area for future research. Various laboratory and clinical evidence have underscored the impact of the gut microbiome on the treatment of cardiovascular disease. For example, the gut microbiome can affect the bioavailability and effectiveness of drugs by metabolizing drug compounds, regulating host genes related to drug transport, and influencing the intestinal reuptake process [171]. Individualized analysis of the microbiome based on metabolomic profiling, holds the potential for guiding dietary choices and optimizing perioperative management [172]. Dietary and nutritional strategies can modify the gut microbiome and accelerate patients' recovery, potentially serving as a complementary approach to enhanced recovery after surgery [173]. Cohort studies and clinical trials are vital for evaluating the therapeutic potential of the microbiome in CHD treatment. This may encompass interventions like probiotic therapy in PICU and strategies to optimize the composition of patients' gut microbiome during the perioperative period. Additionally, innovative approaches like fecal microbiome transplantation have the potential to reshape the gut microbial ecology to produce beneficial metabolites. By maintaining gut microbiome homeostasis, it may be feasible to regulate systemic inflammation resulting from surgical injury or CPB. Reconstituting balanced host‐microbiome interactions in patients with CHD may ameliorate the metabolic and immunity disorder, thus preventing certain clinical outcomes and improving prognosis.

## AUTHOR CONTRIBUTIONS

Shoujun Li provided direction and guidance throughout the preparation of this manuscript. Yuze Liu and Yuan Huang collected and interpreted studies and were major contributors to the writing and editing of the manuscript. Qiyu He, Zheng Dou, Min Zeng, and Xu Wang reviewed and made significant revisions to the manuscript. All authors have read and approved the final manuscript.

## CONFLICT OF INTEREST STATEMENT

The authors declare no conflict of interest.

## Data Availability

This manuscript does not generate any code or data. Supplementary materials (graphical abstracts, slides, videos, Chinese translated version, and update materials) may be found in the online DOI or iMeta Science http://www.imeta.science/.
